# Determinants of maximum cup depth in non-glaucoma and primary open-angle glaucoma subjects: a population-based study

**DOI:** 10.1038/s41433-019-0600-2

**Published:** 2019-09-27

**Authors:** Qing Zhang, Ye Zhang, Chen Xin, Yingyan Mao, Kai Cao, Catherine Jan, Chunyu Guo, Ningli Wang, Ravi Thomas

**Affiliations:** 1 Beijing Institute of Ophthalmology, Beijing Tongren Eye Center, Beijing Tongren Hospital, Capital Medical University, Beijing, China; 2 School of Psychological and Cognitive Sciences, Peking University, Beijing, China; 3 Northwestern University Feinberg School of Medicine, Chicago, USA; 4 Queensland Eye Institute, Brisbane, Australia; 5 University of Queensland, Brisbane, Australia

**Keywords:** Risk factors, Pathogenesis, Optic nerve diseases

## Abstract

**Background/objectives:**

To study the associations of intraocular pressure (IOP) and retinal vessel diameters: central retinal arteriolar equivalent (CRAE) and central retinal venular equivalent (CRVE) with the maximum cup depth (MCD) in subjects with and without POAG.

**Subjects/methods:**

Eligible subjects from the Handan Eye Study. All participants underwent physical and comprehensive eye examinations. Univariable and multivariable linear regression models assessed the association between MCD and other parameters.

**Results:**

Four thousand one hundred and ninety-four eligible nonglaucoma and 40 POAG subjects were analyzed. On univariable analysis, deeper MCD was significantly associated with younger age, male gender, lower systolic blood pressure (BP), higher IOP, higher estimated cerebro-spinal fluid pressure (ECSFP), lower estimated trans-laminal cribrosa pressure difference (ETLCPD), longer axial length, narrower CRAE, narrower CRVE, larger disc area (DA) and a lower prevalence of hypertension and diabetes. On multivariable analysis, significant independent determinants of MCD were larger DA (*P* < 0.001; beta: 0.042; *B*: 0.20; 95% CI: 0.19, 0.22), younger age (*P* < 0.001; beta: −0.09; *B*: −0.002; 95% CI: −0.003, −0.001), higher IOP (*P* < 0.01; beta: 0.040; *B*: 0.003; 95% CI: 0.001, 0.005), and narrower CRAE (*P* < 0.001; beta: −0.06; *B*: −0.001; 95% CI: −0.001, −0.0003). On adding ECSFP and ETLCPD to the model, MCD was associated with IOP but not with estimated CSFP and TLCPD. A 1 μm decrease in CRAE or 1 mmHg increase of IOP was associated with a 1 μm increase of MCD (*P* < 0.001) and 3 μm increase of MCD respectively (*P* = 0.009).

**Conclusions:**

Narrow CRVE and higher IOP are associated with an increase in MCD.

## Introduction

The pathogenesis of primary open angle glaucoma (POAG) remains unclear [1]. The cause is likely to be multifactorial and there is support for both biomechanical as well as vascular theories [2–13].

Published data from population-based studies have reported that narrow retinal vessel diameter, an indicator of impaired autoregulation in the retinal circulation is significantly associated with glaucomatous optic neuropathy (GON) independent of the intraocular pressure (IOP) [8–13]. The Rotterdam Study (RES) and Beaver Dam Study (BDS) did not find an association between narrower retinal vessel caliber and glaucoma [14]. The RES reported that baseline retinal vessel diameters did not influence the risk of incident OAG or incident optic disc changes [15]. The BDS reported that retinal vascular characteristics were associated with hypertension but not with glaucoma [16]. The discrepancy between studies may be due to differences in methodology, statistical analysis, sample characteristics and diagnostic criteria.

Studies on the biomechanical mechanisms have also been published [17–20]. The laminar plate is the primary site of GON [21]. Backward bowing and compression of the laminar plates is associated with the onset and progression of glaucomatous cupping [22]. A recent experimental study showed that monkeys with experimentally reduced CSFP developed morphological changes of the neuro-retinal rim and retinal nerve fiber layer; it did not examine movement of the lamina cribrosa [23]. Clinical studies have reported that POAG and the neuro-retinal rim area (RA) were associated with lower cerebrospinal fluid pressure (CSFP) [17–20]. A lower body mass index (BMI), lower estimated CSFP and a higher estimated trans-lamina cribrosa pressure difference (TLCPD, defined as IOP minus estimated CSFP) were associated with both POAG as well as narrower neuro-retinal RA in three population-based studies [3, 24, 25].

Maximum cup depth (MCD) as determined with confocal scanning laser tomography is defined as the mean depth of the deepest portion of the cup where there is minimum tissue between the lamina cribrosa and the disc surface; movement of this region may be the most representative of the movement of the central lamina cribrosa. To the best of our knowledge, the relationship between the MCD with IOP-related factors (IOP, CSFP, and TLCPD) and the status of retinal blood vessels have not been demonstrated in a population-based study. We report the associations of IOP-related factors and retinal vessels diameters with the MCD as a marker of GON in a population-based setting.

## Methods

The Handan Eye Study (HES) was a population-based survey of participants aged 30 years and above from the Yongnian County, Handan, Heibei Province, China [26]. 6830 of 7557 eligible subjects (90.4% response rate) took part in the study [26, 27].

All subjects underwent a complete ophthalmologic examination including measurement of best corrected visual acuity (BCVA), measurement of IOP with a Perkins hand-held tonometer (KOWA, Nagoya, Japan), slit-lamp biomicroscopy of the anterior segment and dilated stereoscopic bio-microscopy of the fundus. Digital 45° color retinal photography were obtained (TOPCON TRC-NW6S/7S, Tokyo, Japan at the beginning (about 38% of total) and then Canon CR-DGi (Tokyo, Japan (about 62% of total)) [28]. A-scan ultrasound biometry was performed on all subjects (Cine Scan, Quantel Medical, Clemont-Ferrand, France) [26, 27]. All glaucoma suspects and every 10th person underwent visual field testing (standard 24-2 Swedish Interactive Testing Algorithm fast program, Humphrey Visual Field Analyzer 750i; Carl Zeiss, Jena, Germany). Body height and weight were measured and the body mass index (BMI) was calculated. Blood pressure (BP) was measured twice (OMRON Hem-907 blood pressure monitor, OMRON, Kyoto, Japan) after at least 5 min of rest in a sitting position. The average of the two readings was used for analysis. If the difference between two measurements was >10 mmHg for the systolic pressure or 5 mmHg for the diastolic pressure, a third measurement was performed and the average of the three readings was used for analysis. An interviewer-administered questionnaire collected the following information: socioeconomic, lifestyle risk factors, current medications, self-reported history of ocular as well as systemic diseases [26]. Eligible subjects were invited to undergo a comprehensive eye examination, including standardized refraction [29]. Subjective refraction was performed by a trained optometrist for all subjects with vision worse than 20/20 in either eye using a trial frame placed. Auto Refractor-Keratometer (KR8800 Topcon, Tokyo, Japan) readings were used as the starting point for subjective refraction. Refraction data are reported using the subjective refraction when participants had both subjective and objective refraction and autorefraction when only this information was available. Refractive error was reported as spherical equivalent (SE, sum of spherical power and half of the cylinder power, in diopters) [27, 29].

The optic disc was evaluated by slit lamp biomicroscopy: a 90D lens was used to measure the vertical cup disc ratio (VCDR). The border of the optic disc was defined as the inner margin of the peripapillary scleral ring. Standard photographs of optic discs with varying VCDR from 0.2 to 1.0 in 0.1 increments were used for grading. Glaucoma suspects were defined by: IOP > 21 mmHg, VCD ≥ 0.6 (95th percentile of the VCDR in the HES population), asymmetry in the vertical cup-to-disc ratio of ≥0.2, optic disc hemorrhages, retinal nerve fiber layer defects, deposits at the pupil margin consistent with pseudoexfoliation syndrome and pigment deposition on the cornea consistent with pigment dispersion syndrome. All suspects were asked to attend the clinic to undergo perimetry and gonioscopy.

The photographs and clinical notes of all glaucoma suspects were presented to a panel of glaucoma specialists for diagnosis. As described in detail elsewhere, stereoscopic optic disc photographs were evaluated by three glaucoma specialists using a stereoscopic viewer (Screen-Vu Stereoscope; PS Manufacturing, Port-land, OR) [30, 31]. The optic nerve was categorized as “definite glaucoma,” “probable glaucoma,” “possible glaucoma,” or “not glaucoma”. VCDR, notching of the neural rim, localized or diffuse loss of the neural rim, neural rim to disc ratio 0.1, and presence of a nerve fiber layer defect were documented. Cases where consensus was not reached were presented to another panel of glaucoma specialists for final diagnosis [30, 31]. POAG patients had open angles on gonioscopy performed using a Goldman two mirror or a Zeiss 4 mirror lens and did not have a secondary cause of glaucoma [30, 32].

Diabetes mellitus was diagnosed from a self-reported history of medication and/or fasting plasma glucose ≥ 7.0 mmol/L. Hypertension was diagnosed in those providing a history of hypertension on medication or a BP recording ≥ 140/90 mmHg [26, 33].

Optic disc parameters were assessed with the HRT II software (version 1.4.1.0; Heidelberg Engineering, Germany). Imaging was attempted through an undilated pupil with a 15° field of view. Images with significant movement artifact were rejected. If the image quality was poor (standard deviation of more than 30 µm), the pupil was dilated and imaging repeated. A trained operator masked to study findings drew the contour line around the inner margin of the peripapillary scleral ring. The protocol for HRT II examination is detailed elsewhere [34]. Disc area (DA) and maximum cup depth (MCD) were automatically calculated by the HRT II software [34]. MCD represents the average depth of the deepest portion of the cup. HRTII was not used in the diagnosis of glaucoma.

Retinal arteriolar and venular diameters were measured with a computer-assisted program (IVAN; University of Wisconsin, Madison, WI, USA) using a standardized protocol [35]. A trained grader masked to participant characteristics performed all vessel measurements in the right eye of each participant. All arterioles and venules in a zone between 0.5 and 1 disc diameters from the optic disc margin were measured. The average arteriolar and venular caliber was designated the central retinal arteriolar equivalent (CRAE) and central retinal venular equivalent (CRVE), respectively. Details of the digital image preparation are described elsewhere [35].

The CSFP was calculated using the formula: CSFP (mmHg) = 0.44 × body mass index (kg/m^2^) + 0.16 × diastolic BP (mmHg)–0.18 × age (years)−1.91. TLCPD was calculated as IOP minus the calculated CSFP. This formula was used in three population-based studies [24, 36, 37].

The analysis was restricted to eyes of non-glaucomatous and POAG subjects in whom gradable retinal photographs and HRTII images of acceptable quality could be obtained. Highly myopic eyes, defined as a myopic refractive error of ≥−8 diopters or axial length ≥ 26.5 mm were excluded [3, 38, 39].

### Statistical analysis

Data analysis was performed using a commercially available software program (SPSS version 21.0; IBM-SPSS Inc., Chicago, IL, USA). The right eye of eligible participants was used for analysis. Initially, *t*-test and *χ*^2^*-*test were used to compare differences in the parameters (age, gender, axial length) between those included and excluded. Next, univariable and multivariable linear regression models were used to assess the association between MCD and other parameters (continuous parameters: age, ocular parameters, systemic parameters; binary variables: gender, hypertension, diabetes). This was first done for the entire group and then for the non-glaucomatous group and the POAG group separately. Durbin–Waston value was calculated to check if the dependent variable was suitable for linear regression analysis. Variance inflation factor was calculated to measure multicollinearity in the variables. All *P*-values are two sided and *P* < 0.05 was considered statistically significant. Propensity score matching detailed in a previous study was used to determine the differences in characteristics between those with and without POAG [3].

## Results

**Fig. 1:**
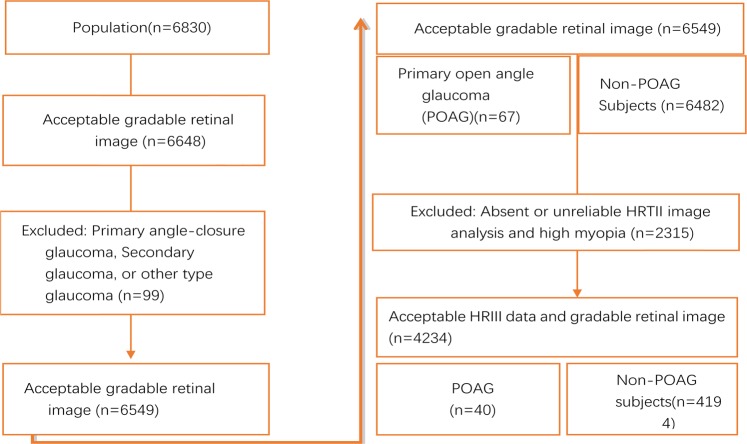
Subject selection and reasons for exclusion

6648 (97.3%) of 6830 study participants individuals had gradable fundus photographs. 4532 (66.4%) individuals, [2030, (44.8%) men] without glaucoma (4487) and 45 POAG subjects had both gradable fundus photographs and HRT images of good quality. After excluding 298 highly myopic eyes, 40 POAG and 4194 non-POAG subjects (4234; 63.7% of those eligible) were available for final analysis (Fig. 1). Compared to those excluded, the 4234 included were younger, had a higher BMI, a higher prevalence of hypertension, higher estimated CSFP and IOP, lower TLCPD, higher CRAE and CRVE, a lower proportion of males and a lower prevalence of diabetes. There were no significant differences in axial length, disc area, maximum cup depth, waist-to-hip ratio or prevalence of POAG (Table 1).

**Table 1:** Characteristics of included and excluded participants

	Inclued (*n* = 4234)	Excluded (*n* = 2596)	*P*-value
Age (years)	51.12 ± 10.80	54.31 ± 13.83	<0.001
SBP (mmHg)	139.11 ± 21.74	137.82 ± 23.65	0.026
DBP (mmHg)	78.14 ± 12.16	76.07 ± 11.99	<0.001
BMI	24.64 ± 3.65	24.24 ± 3.96	0.002
W/H ratio	0.90 ± 0.06	0.90 ± 0.10	0.969
CSFP (mmHg)	12.26 ± 3.29	11.38 ± 3.64	<0.001
IOP (mmHg)	15.11 ± 2.80	14.84 ± 3.29	0.001
TLCPD (mmHg)	2.86 ± 3.67	3.23 ± 4.32	0.001
AL (mm)	22.79 ± 0.76	22.86 ± 1.2	0.056
DA (mm^2^)	2.24 ± 0.43	2.26 ± 0.40	0.379
CRAE (μm)	157.39 ± 22.06	150.38 ± 31.62	<0.001
CRVE (μm)	240.87 ± 33.02	228.44 ± 38.74	<0.001
AVR	0.66 ± 0.07	0.66 ± 0.13	0.002
Maximum cup depth (mm)	0.58 ± 0.22	0.58 ± 0.21	0.936
Gender (male%)	1882 (44.4%)	1281 (49.3%)	<0.001
Diabetes (%)	237 (6.2%)	150 (8.5%)	0.001
Hypertension (%)	2057 (48.6%)	1140 (46.1%)	0.05
POAG (%)	40 (0.9%)	27 (1.1%)	0.61

Continuous data are represented as mean  ±  SD; and categorical data are represented as *n* (%) for overall. Comparisons between groups were performed with independent-samples *t*-test for continuous data, and Pearson’s Chi-square test or Fisher’s exact test for categorical data

*SBP* systolic blood pressure, *DBP* diastolic blood pressure, *IOP* intraocular pressure, *AL* axial length, *DA* disc area, *CRAE* central retinal arteriolar equivalent, *CRVE* central retinal venular equivalent, *AVR* CRAE/CRVE ratio, *W/H ratio* Waist-to-hip ratio, *BMI* body-mass-index, *POAG* primary open-angle glaucoma, *CSFP* estimated cerebro-spinal fluid pressure, *TLCPD* lower estimated trans-laminal cribrosa pressure difference

*P* < 0.05, indicated a significant difference found between the included group and the excluded group

On univariable analysis (Table 2), deeper MCD was significantly associated with the following systemic and ocular parameters: younger age, male gender, lower mean systolic BP, higher IOP, higher estimated CSFP, lower TLCPD, longer axial length, narrower CRAE, narrower CRVE and larger disc area (DA) and a lower prevalence of hypertension and diabetes. It was not significantly associated with mean diastolic BP, BMI, waist-to-hip ratio or CRAE/CRVE ratio (AVR).

**Table 2:** Univariable analysis of the associations between the maximum cup depth (mm) and ocular and systemic parameters of the Handan eye study (40 POAG and 4194 non-glaucoma)

Parameters	*P*-value	Coefficient *B*	95% CI of *B*
Age (years)	<0.001	−0.003	−0.004, −0.003
AL (mm)	<0.001	0.025	0.017, 0.033
IOP (mmHg)	0.006	0.003	0.001, 0.006
TLCPD (mmHg)	0.006	−0.003	−0.004, −0.001
CSFP (mmHg)	<0.001	0.006	0.004, 0.008
DA (mm^2^)	<0.001	0.210	0.210, 0.236
CRAE (μm)	<0.001	−0.001	−0.001, 0.000
CRVE (μm)	0.003	−0.0003	0.000, 0.000
SBP (mmHg)	**<0.001**	**−0.001**	**−0.001, −0.001**
DBP (mmHg)	0.800	<0.001	0.000, 0.001
AVR	0.340	−0.046	−0.143, −0.050
W/H ratio	0.170	0.080	−0.036, 0.199
BMI (kg/m^2^)	0.150	−0.001	−0.003, 0.000
*Gender*^a^			
Female	<0.001	−0.044	−0.057, −0.031
Male			
*Diabetes*^a^			
No			
Yes	0.072	−0.026	−0.053, 0.002
*Hypertension*^a^			
No			
Yes	<0.001	−0.032	−0.045, 0.018

*P*-value = statistical significance of the association

CI = confidence interval

Coefficient β = Regression Coefficient (un-standardized Beta)

*SBP* systolic blood pressure, *DBP* diastolic blood pressure, *IOP* intraocular pressure, *AL* axial length, *DA* disc area, *CRAE* central retinal arteriolar equivalent, *CRVE* central retinal venular equivalent, *AVR* CRAE/CRVE ratio, *W/H ratio* Waist-to-hip ratio, *BMI* body-mass-index

^a^Note: Gender, diabetes, hypertension was analyzed by mixed linear model

For multivariable analysis, Model 1 included all parameters demonstrating a significant association with MCD in the univariable analysis. After step-wise dropping of parameters that were no longer significantly associated with MCD, we arrived at a model in which determinants of the higher MCD were a larger DA, younger age, male gender, higher IOP, diagnosis of POAG and narrower CRAE. A standard deviation (SD) decrease of CRAE and increase of IOP were associated with an increase of MCD in a ratio of 1:0.06 and 1:0.04, respectively (Table 3). Model 2 included the same independent variables as Model 1 as well as the estimated CSFP and TLCPD. A higher IOP was again found to be significantly associated with a deeper MCD, while estimated CSFP or TLCPD was not (Table 3).

**Table 3:** Multivariable analysis of the determinants of the Maximum Cup Depth of the Handan Eye Study (40 POAG and 4194 non-glaucoma)

Parameter	*P*-value	Regression coefficient *B*	Standardized regression Coefficient	95% Confidence interval for *B*	Variance inflation factor
*Model 1*					
DA (mm^2^)	<0.001	0.20	0.42	0.19, 0.22	1.05
Gender	<0.001	−0.04	−0.08	−0.05, −0.02	1.03
Age (years)	<0.001	−0.002	−0.09	−0.003, −0.001	1.07
Presence of POAG	<0.001	0.17	0.08	0.11, 0.23	1.02
CRAE (μm)	<0.001	−0.001	−0.06	−0.001, −0.0003	1.01
IOP (mmHg)	0.009	0.003	0.04	0.001, 0.005	1.03
*Model 2*					
DA (mm^2^)	<0.001	0.20	0.41	0.19, 0.22	1.05
Age (years)	<0.001	−0.002	−0.10	−0.003, −0.001	1.07
Gender	<0.001	−0.04	−0.09	−0.05, −0.03	1.03
Presence of POAG	<0.001	0.17	0.07	0.10, 0.24	1.02
CRAE (μm)	<0.001	−0.001	−0.06	−0.001, −0.0003	1.01
IOP (mmHg)	0.005	0.003	0.04	0.001, 0.005	1.03

Model 1: adjusted for the DA, AGE, GENDER, SBP, AL, IOP, CRAE, CRVE, HYPERTENSION, DM, POAG. The Durbin–Watson value: 1.98

Model 2: adjusted for the same variables in model 1 plus estimated CSFP and TLCPD. The Durbin–Watson value: 1.99

*DA* disc area, *CRAE* central retinal arteriolar equivalent, *IOP* intraocular pressure

The associations found in both univariable and multivariable analyses also remained largely unchanged in persons without evidence of clinical glaucoma: the determinants of MCD were DA, age, gender, CRAE, and IOP (*n* = 4194, data not shown).

The analysis was repeated in POAG subjects: the MCD was not significantly associated with any other parameters studied in the univariable and multivariable analysis.

**Fig. 2:**
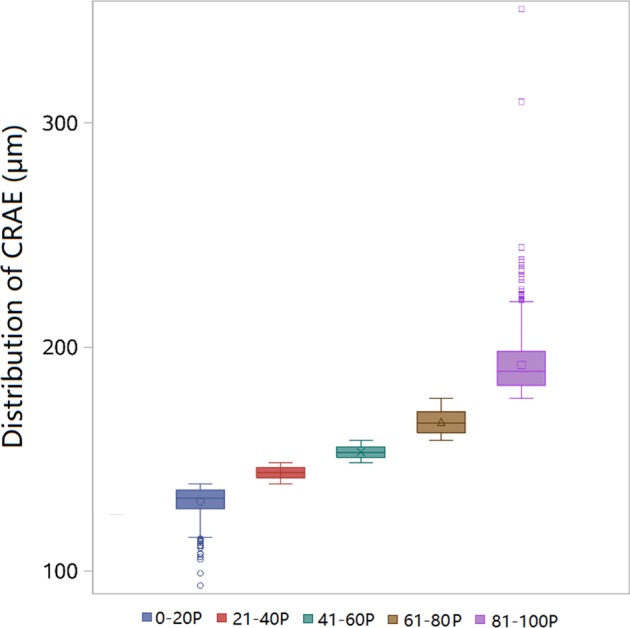
The boxplot of distribution of CRAE by different percentiles. *P* stands for percentiles

Figure 2 plots the average (and 95% CI) by quintiles of CRAE. There is a linear trend across the quintiles.

## Discussion

Our findings are in agreement with previous population-based studies that found an association between narrower retinal arteriolar changes with GON independent of IOP [8, 9, 11]. Furthermore, these associations remained largely unchanged in persons without evidence of clinical glaucoma [11, 12]. The RES and BDS did not adjust for optic disc area in the analysis and did not find an association between narrower retinal vessel caliber and glaucoma [14–16]. The Singapore Malay Eye Study and ours found an association between narrower retinal vessel caliber and rim area after adjusting for optic disc area in the analysis [3, 12]. Characteristics associated with large discs include a larger number of nerve fibers, more and larger lamina cribrosa pores, a larger neuro-retinal rim area, and a larger cup disc ratio [40]. As size of the neuro-retinal rim and cup are often used in the differential diagnosis of glaucoma, it is important to estimate disc size in order to make an accurate assessment [40]. The BDS reported that small optic discs were associated with smaller central retinal artery and central retinal vein diameter [10, 41]. The BMES reported that generalized retinal arteriolar narrowing is significantly associated with optic nerve damage caused by OAG as well as with long-term risk of OAG; optic disc area was not adjusted in the analysis [9, 10]. Potential explanations for the discrepancy between the BMES and the RES may include differences in follow-up duration, sample characteristics, and analysis [10].

We found that retinal arteriolar caliber had a stronger association with MCD than retinal venules. This may support the notion that the movement of the central lamina cribrosa may be associated with the insufficiency of the blood supply. However, our results are only associations and cannot explain pathogenesis.

The association of age and gender with HRT parameters in the included subjects should be interpreted with caution. However, the significant correlation between age, gender, and MCD indicates the importance of adjusting for both systemic parameters as a possible confounder in multiple regression analyses.

A histological study has reported that the micro-vascular bed of the disc is an integral part of the retina-optic nerve vascular system [42]. A clinical study reported that the MCD was significantly larger in glaucomatous eyes compared to non-glaucomatous eyes [43]. Considering that CRAE is a part of the retina-optic nerve vascular system, the association between deeper MCD and narrower CRAE in the population suggests a relationship between altered optic nerve head (ONH) blood flow and movement of the lamina region. If the ONH is considered as a load-bearing structure, several etiological factors are implicated in glaucomatous ONH damage [44, 45]. Insufficiency of the arterial blood supply to the laminar region could induce cell-mediated connective tissue changes that would weaken the laminar beams making them more prone to failure under previously safe levels of load-related mechanical stress resulting in deeper MCD [4, 46, 47]. From this point of view, the pathophysiology of glaucomatous damage in the ONH likely involves a change in both the IOP-related pressure, as well as insufficient blood supply to the laminar region. Our finding that higher MCD was associated with higher IOP and retinal vessel caliber (narrow CRAE) in a population setting support this notion.

When we adjusted the same independent variables as in model 1 but not IOP, estimated CSFP or TLCPD, a higher TLCPD (standardized coefficients beta = 0.043, *P* = 0.007, CI = 0.001,0.004) was again found to be significantly associated with a smaller MCD, while estimated CSFP was not (data not shown). IOP is part of the formula used to calculate TLCPD; this may be the reason why TLCPD was not associated with MCD in the multivariable model 2 while IOP was; IOP seems to be the determinant factor here. In a previous report in the same population, after adjusting for other confounding factors in the multivariable analysis narrower rim area was significantly associated with lower CSFP or higher TLCPD. Our findings are similar to the report that primary changes in CSFP or TLCPD are not likely to lead to laminar deformation or to produce a “glaucomatous” optic neuropathy in which the lamina deforms [23, 48–55].

The strengths of our study are the population-based design and quantification of variables included in the statistical analysis.

There are several limitations. First, as in any population-based investigation, non-participation can lead to selection bias. 4234 (63.7%) subjects had acceptable retinal photographs and HRTII images for analysis. While participation rates compare favorably with other large population-based studies, we cannot exclude the possibility that if photographs of all study participants were included the results might have been different.

Second, due to the cross-sectional nature of our data we cannot determine whether vascular changes reflect a primary ischemic pathologic process inducing cell-mediated connective tissue changes leading to the backward bowing of the laminar plates or result from autoregulatory changes in retina-optic nerve vascular system secondary to remodeling of the ECM in the laminar region.

Third, the CRAE is the mean value of the retinal blood vessel diameter; it only reflects general changes of the blood vessels in the optic disk and retina and cannot indicate the site or level of changes. A further limitation is that MCD was not significantly associated with IOP and vascular factors in the univariable and multivariable analysis in POAG. The small number of POAG cases probably precluded the detection of real associations. An earlier study in the same population reported that compared to 67 age-matched and gender-matched controls, the mean RA was significantly lower and the mean MCD, mean IOP, and the mean TLCPD significantly higher in 67 POAG subjects (*P* ≤ 0.001) [3]. The median CRAE and CRVE was significantly lower and mean MCD, mean IOP, and mean TLCPD was significantly higher in the 125 POAG subjects diagnosed using the ISGEO definition compared to the 125 age and gender-matched control non-glaucoma subjects (*P* ≤ 0.05) [3]. Finally, the morphological change in the lamina cribrosa was defined by HRTII; future studies may address this deformation using three-dimensional histomorphometry and longitudinal swept-source OCT imaging.

In summary, in a population-based setting, a deeper MCD was associated with higher IOP and a narrower CRAE. As MCD is a marker for glaucomatous optic nerve damage, our findings support the view that both IOP change and insufficiency of blood supply in tissue deformation of the laminar region.

## Summary

### What was known before

- The pathogenesis of primary open angle glaucoma (POAG) remains unclear.
- The laminar plate is the primary site of glaucomatous optic neuropathy. The backward bowing and compression of the laminar plates is associated with the onset and progression of glaucomatous cupping.
- Maximum cup depth (MCD) is defined as the mean depth of the deepest portion of the cup where there is minimum of tissue between the lamina cribrosa and the disc surface; movement of this region best reflects the movement of the central lamina cribrosa.

### What this study adds

- Question: What are the association of changes in the retinal circulation, biomechanical changes at the optic disc, and displacement of the lamina cribrosa (LC) in a rural adult Chinese population?
- Findings: In a cross-sectional study of 4234 subjects, an increase in intraocular pressure (IOP) and narrowing of the central retinal arteriolar were found to be associated with increased maximum cup depth (MCD).
- Meaning: Our findings of a population-based study provide important evidence to the theory that glaucomatous optic neuropathy originates from both high IOP and insufficiency of blood supply. The standard deviation (SD) decrease of central retinal arteriolar equivalent (CRAE) and increase of IOP were associated with the increase of MCD in a ratio of 1:0.06 and 1:0.04, respectively.
